# Association between genetic ancestry and *EDAR* rs3827760 genotypes in Latin American populations

**DOI:** 10.7705/biomedica.7693

**Published:** 2026-06-01

**Authors:** Jorge Briceño, Ángel Roco-Videla, Germán Manríquez-Soto, Sergio V. Flores

**Affiliations:** 1 Centro de Análisis Cuantitativo en Antropología Dental, Instituto de Investigación en Ciencias Odontológicas, Facultad de Odontología, Universidad de Chile, Santiago, Chile Universidad de Chile Instituto de Investigación en Ciencias Odontológicas Facultad de Odontología Universidad de Chile Santiago Chile; 2 Facultad de Ingeniería, Universidad Católica de la Santísima Concepción, Concepción, Chile Universidad Católica de la Santísima Concepción Facultad de Ingeniería Universidad Católica de la Santísima Concepción Concepción Chile; 3 Departamento de Antropología, Facultad de Ciencias Sociales, Universidad de Chile, Santiago, Chile Universidad de Chile Departamento de Antropología Facultad de Ciencias Sociales Universidad de Chile Santiago Chile; 4 Facultad de Ciencias de la Salud, Universidad Católica Silva Henríquez, Santiago, Chile Universidad Católica Silva Henríquez Facultad de Ciencias de la Salud Universidad Católica Silva Henríquez Santiago Chile; 5 Vicerrectoría de Investigación, Universidad Arturo Prat, Santiago, Chile Universidad Arturo Prat Vicerrectoría de Investigación Universidad Arturo Prat Santiago Chile

**Keywords:** Receptors, ectodysplasin, polymorphism, single nucleotide, genotype, Latin American, receptores de la ectodisplasina, polimorfismo de nucleótido simple, genotipo, América Latina

## Abstract

**Introduction.:**

The *EDAR* (ectodysplasin A receptor) gene is associated with the development of ectodermal structures. The rs3827760 variant is highly frequent in East Asian and Native American populations, and this study analyzes its relationship with genetic ancestry proportions in a Latin American population sample.

**Objective.:**

To analyze how genetic ancestry proportions influence the distribution of single nucleotide polymorphism (SNP) rs3827760 genotypes of the *EDAR* gene in Latin American populations.

**Materials and methods.:**

Genetic ancestry proportions were estimated using a panel of 446 ancestry-informative SNPs, applying the STRUCTURE program and univariate and multivariate logistic regression models.

**Results.:**

Native American ancestry proportions showed a significant positive association with the prevalence of rs3827760 GG genotypes, while European ancestry showed a negative association.

**Conclusion.:**

Native American and European genetic ancestry proportions influence the distribution of SNP rs3827760 genotypes of the *EDAR* gene in Latin American populations.

## Resumen

**Introducción.:**

El gen *EDAR* (*ectodysplasin A receptor*) se asocia con el desarrollo de las estructuras ectodérmicas. La variante rs3827760 presenta una alta frecuencia en poblaciones asiáticas y nativas americanas; este estudio analiza su relación con proporciones de ascendencia genética en una muestra de población latinoamericana.

**Objetivo.:**

Analizar cómo las proporciones de ancestro genético influyen en la distribución de los SNP de los genotipos de rs3827760 de *EDAR* en las poblaciones latinoamericanas.

**Materiales y métodos.:**

Se estimaron las proporciones de ancestro genético utilizando un panel de 446 SNPs informativos de ancestro, aplicando el programa STRUCTURE y modelos de regresión logística univariados y multivariados.

**Resultados.:**

Las proporciones de ancestro nativo americano mostraron una asociación positiva significativa con la prevalencia de los genotipos GG del SNP rs3827760, mientras que el ancestro europeo mostró una asociación negativa.

**Conclusión.:**

Las proporciones de ancestro nativo americano y europeo influyen en la distribución de los genotipos del SNP rs3827760 de *EDAR* en las poblaciones latinoamericanas.

The *EDAR* (ectodysplasin A receptor) gene is associated with the development of ectodermal structures such as hair, teeth, and sweat glands. This gene encodes a receptor from the tumor necrosis factor (TNF) family, which, upon activation through binding to its ligand ectodysplasin (EDA), triggers a signaling cascade via the activation of the NF-kB transcription factor, essential for the normal development of these structures ^1^.

Mutations in *EDAR*, particularly in its “death domain”, can affect the interaction with the EDARADD protein, resulting in ectodermal dysplasias characterized by the loss or malformation of dermal structures ^2^^,^^3^. This domain is highly conserved, highlighting the importance of *EDAR* in tissue formation and differentiation during human development ^4^.

The SNP rs3827760 in the *EDAR* gene encodes a valine-to-alanine substitution at position 370 (EDAR.Val370Ala), increasing receptor signaling and leading to significant phenotypic changes, such as thicker and straighter hair, significant modifications in dental shape (i.e., incisors shovelling), and higher sweat gland density ^3^. This variant, present at very high frequencies in East Asian and Native American populations, has been studied for its impact on phenotypic variability and its association with distinctive physical characteristics ^5^^,^^6^. Additionally, it has been suggested that this gain of receptor function may have provided an adaptive advantage in specific environments ^3^.

The SNP rs3827760 has shown clear evidence of positive selection in East Asian populations, where it reaches frequencies close to 90% in some groups ^3^. This high level of selection has been associated with enhanced NF-kB signaling, which may have conferred adaptive advantages in the cold and dry climates of these regions, where traits such as higher sweat gland density and thicker hair could have improved environmental protection ^5^^,^^7^. Haplotype studies have demonstrated that this variant rapidly spread in some populations due to natural selection ^3^^,^^7^.

The derived allele of rs3827760 shows a marked geographical distribution, predominantly in East Asia and Native America, but is almost nonexistent in Europe and Africa ^3^. This distribution suggests that the variant emerged and expanded under specific selective pressures in these regions ^6^.

In Latin American populations, the variability of rs3827760 is influenced by genetic admixture with Native American ancestors, who carry the derived allele at high frequencies ^8^. In contrast, in populations with greater European or African ancestry, the frequency of this allele is significantly lower, reflecting the migratory history and admixture of these populations ^9^, and showing how the variability of rs3827760 in Latin America reflects the complex demographic history of the region ^3^.

This study analyzes the influence of genetic ancestry proportions on the distribution of rs3827760 genotypes in the *EDAR* gene within a Latin American population sample. To our knowledge, this relationship has not been previously examined. Using statistical analyses, we investigate the association between this genetic variant and the diverse ancestral components present in the region, generating data relevant to both population genetics and forensic applications.

By addressing this gap, the present study contributes novel evidence on how continental ancestry components shape the distribution of functional variants in Latin America. These findings may refine interpretations in population genetics research and support ancestry inference strategies in forensic and anthropological applications.

## Materials and methods

The estimation of genetic ancestry proportions was carried out using a panel of 446 ancestry-informative SNP, following the method described by Galanter *et al*. ^10^. This panel allows the identification of the contribution of five human macro-populations: African, East Asian, South Asian, European, and Latin American. Genotypic data for the SNP rs3827760 in the Latin American population were downloaded from the 1000 Genomes Project database, specifically selecting a sample consisting of 347 individuals.

All available Latin American subpopulations from phase 3 of the 1000 Genomes Project were included [Colombians from Medellín, Mexicans from Los Angeles, Peruvians from Lima, and Puerto Ricans from Puerto Rico; (N = 347)], applying only the standard quality control filters of the project (call rate ≥ 98% and Hardy-Weinberg equilibrium with p > 1 × 10^-6^). No additional exclusion criteria were applied.

Genetic ancestry proportions were modeled using the STRUCTURE program ^11^^,^^12^, which employs a Bayesian approach to infer population structure from genotypic data. The normality of ancestry distributions was assessed using the Kolmogorov-Smirnov test due to the considerable sample size. Upon confirming that the distributions did not follow a normal distribution (p < 0.05), non-parametric Kruskal-Wallis and Wilcoxon tests were applied to evaluate the association between ancestry proportions and the SNP rs3827760 genotypes of the *EDAR* gene.

To assess the association between ancestry proportions and the probability of carrying EDAR phenotype-determining genotypes (GA or GG), univariate and multivariate logistic regression analyses were performed. In the univariate models, each ancestry proportion (Native American, European, and African) was individually evaluated as a predictor of the EDAR phenotype determining genotypes. Subsequently, a multivariate model including the three ancestry proportions simultaneously was conducted to determine their combined effect on predicting these genotypes.

The Ridge regression technique was used to adjust the multivariate model and evaluate potential collinearity issues among ancestry variables. The model’s performance was evaluated by estimating the area under the curve of the receiver operating characteristic (ROC).

We analyzed individual genetic ancestry proportions (Native American, African, and European) using principal component analysis in Python. This analysis was performed with the scikit-learn library on the three ancestry variables, and the first two components were retained.

Genotype data for the EDAR SNP were used to classify individuals into three groups (AA, AG, GG), with AG and GA treated as equivalent. The principal component analysis results were visualized using matplotlib, and differences across genotypes were assessed through one-way ANOVA using scipy.stats.

The Kruskal-Wallis and Wilcoxon rank-sum tests were selected due to the non-normal distribution of ancestry proportions, as indicated by the Kolmogorov-Smirnov test. These non-parametric tests are suitable for comparing continuous variables across genotype groups when parametric assumptions are not met. Logistic regression models were applied to evaluate the association between ancestry proportions and the probability of carrying specific EDAR genotypes. This approach allows estimation of effect sizes (odds ratios) while adjusting for multiple predictors in both univariate and multivariate contexts.

**Figure 1.**
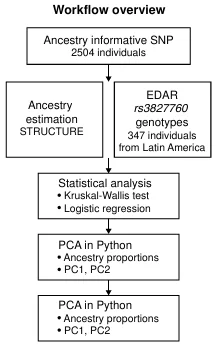
Workflow overview of the analytical pipeline

In addition, principal component analysis was used to summarize ancestry variation across individuals and reduce dimensionality. A oneway ANOVA was then used to assess whether variation along the first two principal components differed significantly by genotype, taking advantage of principal component analysis orthogonality and ANOVA’s sensitivity to group differences along continuous axes. Figure 1 shows the workflow of the analytical pipeline.

## Results

This study analyzed the distribution of SNP rs3827760 genotypes in the *EDAR* gene concerning Native American, European, and African genetic ancestry proportions. The descriptive analysis results showed that the median proportion of Native American ancestry was higher in individuals with the GG genotype (0.684), followed by those with the GA (0.272) and AA (0.071) genotypes.

Conversely, the median proportion of European ancestry was higher in individuals with the AA genotype (0.563), decreasing in GA (0.4385) and GG (0.1380) genotypes. Regarding African ancestry, the medians were lower across all genotypes, with the highest in individuals with the AA genotype (0.0175), followed by GA (0.0180) and GG (0.0040).

The Kruskal-Wallis analysis revealed statistically significant differences in Native American, European, and African ancestry proportions (p < 0.001) among rs3827760 genotypes. *Post-hoc* Wilcoxon tests with Holm correction confirmed differences between all genotype pairs for Native American and European ancestry proportions (p < 0.001), while for African ancestry, significant differences were mainly observed between the GG genotype and the other two.

In the univariate models, a higher proportion of Native American ancestry was associated with an increased probability of carrying EDAR genotypes (OR = 5.11, 95% CI: 3.91-6.69, p < 0.001). In contrast, a higher proportion of European ancestry was associated with a lower probability of carrying EDAR genotypes (OR = 0.19, 95% CI: 0.11-0.29, p < 0.001), as was African ancestry, although with a less pronounced effect (OR = 0.02, 95% CI: 0.00 0.57, p = 0.0128).

**Figure 2.**
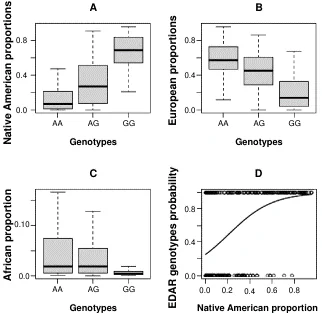
Distribution of genetic ancestries by genotype and logistic regression model prediction between Native American ancestry proportion and the probability of *EDAR* genotypes. A, B, and C: Distribution of genetic ancestries by genotype. D: Logistic regression model prediction between the proportion of Native American ancestry and the probability of genotypes associated with the *EDAR* phenotype.

In the multivariate model, which simultaneously included the three ancestry proportions, it was observed that the proportion of Native American ancestry maintained a significant positive association with the risk of carrying EDAR genotypes (OR = 3.53, 95% CI: 2.00-4.96, p = 5.97e-06). The proportion of European ancestry maintained a significant negative association (OR = 0.06, 95% CI: 0.03-0.25, p = 0.00093), while the proportion of African ancestry was not statistically significant in this model (OR = 0.14, 95% CI: 0.01-2.71, p = 0.247). Figure 2 shows the results of genetic ancestry distributions by genotype and the logistic regression model between the Native American ancestry proportion and the probability of EDAR genotypes.

The evaluation of the multivariate model fit using Ridge regression analysis yielded an area under the curve of 0.8135, indicating good predictive power.

**Figure 3.**
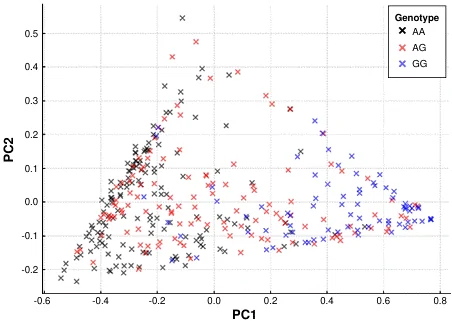
Individual genetic ancestry proportions (Native American, African, and European) using principal component analysis

In the principal component analysis (figure 3), PCI accounted for most of the variance and reflected an ancestry gradient from Native American (positive loading) to European (negative loading). The second component (PC2) showed mixed loadings without a clear interpretive axis. A statistically significant difference in PCI values was observed among EDAR genotypes (p < 0.0001), while PC2 showed no significant differences (p = 0.465).

## Discussion

The results of this study evidence a significant association between genetic ancestry proportions and genotypes determining the EDAR phenotype, specifically the SNP rs3827760. A higher proportion of Native American ancestry was associated with a greater prevalence of the GG genotype, while a higher proportion of European ancestry was associated with a greater prevalence of the AA genotype. These findings are consistent with previous studies that have identified a high frequency of the derived allele rs3827760 in East Asian and Native American populations, in contrast to its low prevalence in European and African populations ^6^^,^^8^.

Given that the derived G allele has been functionally linked to phenotypic traits such as increased hair thickness, shovel-shaped incisors, and greater density of eccrine sweat glands, the differential distribution observed in our sample may also have implications for the expression of these traits in admixed Latin American populations.

The strong positive selection acting on SNP rs3827760 in East Asia could explain its high frequency in Native American populations, as these populations largely descend from Asian ancestors ^5^. This phenomenon of positive selection has been widely documented in genomic studies, where variants conferring adaptive advantages under specific conditions rapidly spread within a population ^7^^,^^3^.

While some studies have proposed adaptive advantages related to mammary duct branching and vitamin D transfer through breast milk ^13^, a recent discussion has critically challenged this hypothesis, highlighting contradictory evidence on breast density and vitamin D levels in carriers of the allele ^14^. These findings emphasize the need for caution when interpreting the adaptive role of EDAR variants in human populations.

On the other hand, the lower frequency of the derived allele in populations with a higher proportion of European ancestry could be related to the absence of similar selective pressures in Europe. In this context, studies like that of Bryk *et al*. suggest that the variability in rs3827760 frequency across different regions of the world reflects the unique evolutionary history of each population, influenced by specific environmental and demographic factors ^9^.

It is also important to note that, although a significant association was found between ancestry proportions and genotypes determining the EDAR phenotype, the multivariate model showed that African ancestry was not a significant predictor in this context. This could be due to the low frequency of the variant in African populations, as previously reported by the 1000 Genomes Project ^15^. However, this finding underscores the complexity of genetic interactions and the need for additional studies with larger and more diverse samples to confirm these observations.

Although the sample size was sufficient for the analyses performed -as indicated by the strong statistical associations found and the good predictive power of the model (AUC = 0.81)- it does not represent all the genetic diversity found in Latin America. The data come from a limited number of populations included in the 1000 Genomes Project, which may not reflect the variation present in other regions or groups. These results can serve as a basis for future studies focused on specific Latin American populations, using local samples to better understand the relationship between ancestry and genotype.

Principal component analysis confirmed the association between EDAR SNP variation and individual genetic ancestry, particularly along the Native American-European axis. The observed differentiation aligns with known population-level patterns of EDAR allele frequencies in the Americas, where the ancestral allele is more prevalent among individuals with higher European ancestry. The lack of differentiation in PC2 supports that the observed association is restricted to the main axis of genetic variation between populations.

While the study accounts for potential collinearity among ancestry proportions using Ridge regression, other confounding factors may influence the observed associations. Population stratification and differences in admixture timing between subpopulations could affect both ancestry estimates and genotype distributions. Although our analyses focus on Latin American individuals from the 1000 Genomes Project and treat them as a single admixed group, heterogeneity in demographic histories could introduce subtle biases. Future studies incorporating local ancestry inference or modeling of admixture dynamics may help further refine these associations.

Although this study focuses on genetic ancestry, it is important to note that the high frequency of the rs3827760 derived allele in Latin American populations likely reflects its strong positive selection in East Asia prior to the peopling of the Americas. After the initial migration, the variant would have been carried by ancestral Native American groups, rather than being subject to renewed selective pressures in the Americas. Nonetheless, exploring whether environmental factors may have contributed to the retention of this allele remains an open question for future research.

Beyond its relevance for population genetics, this finding may also have applications in forensic genetics. Given the differential distribution of the rs3827760 variant across ancestral components, particularly Native American and European ancestries, it may contribute to improving ancestry inference in admixed populations.

In conclusion, our results support the hypothesis that Native American and European genetic ancestry proportions influence the distribution of genotypes determining the EDAR phenotype in mixed Latin American populations. This study contributes novel data on the distribution of rs3827760 genotypes in relation to genetic ancestry proportions in Latin American populations, a relationship that had not been previously explored.

By highlighting how Native American and European ancestries influence the presence of this variant, our findings provide a foundation for future research on population differentiation and admixture. In forensic genetics, understanding the ancestry-related distribution of informative SNPs such as rs3827760 can improve individual profiling and ancestry inference in admixed populations, offering practical applications in both anthropological and legal contexts.
